# The dual role of extracellular vesicles derived from animal and human immune cells: A systematic review

**DOI:** 10.5455/javar.2025.l915

**Published:** 2025-06-02

**Authors:** Joseph Bagi Suleiman, Norhayati Liaqat Ali Khan, Maryam Azlan

**Affiliations:** 1 School of Health Sciences, Universiti Sains Malaysia, Kubang Kerian, Kelantan, Malaysia; 2 Department of Science Laboratory Technology, Akanu Ibiam Federal Polytechnic, Unwana, Nigeria; 3 Centre for Preclinical Science Studies, Faculty of Dentistry, Universiti Teknologi MARA (UiTM), Sungai Buloh, Malaysia; 4 Cardiovascular Advancement and Research Excellence Institute (CARE Institute), Universiti Teknologi MARA, Selangor, Malaysia

**Keywords:** B cell, extracellular vesicles, immune cells, monocyte, neutrophil, T cell

## Abstract

This review aims to examine the functions of extracellular vesicles (EVs) originating from animal and human immune cells, with a focus on their roles in immunomodulation and therapeutic potential. It highlights their dual effects in infection and autoimmunity, cancer treatment, inflammatory conditions, and regenerative medicine while also addressing the challenges in standardizing EV production, isolation, and characterization for clinical applications. This review highlights the need for robust protocols to advance EV-based therapies. It also synthesizes current literature on immune cell-derived EVs, with a focus on their mechanisms of action in intercellular communication, immune modulation, and therapeutic delivery. Additionally, it examines studies that explore the regenerative potential of immune cell-derived EVs and discusses the technical and methodological challenges involved in EV research and clinical translation. EVs from immune cells can either boost or reduce immune responses in tumor therapy, which greatly affects how cancer develops and how well treatments work. These EVs also show promise in managing inflammatory diseases through immune modulation and targeted therapeutic delivery. Furthermore, immune cell-derived EVs possess regenerative properties, contributing to tissue repair and the maintenance of homeostasis. Despite these promising roles, challenges related to the standardization of EV production, isolation, and characterization continue to impede clinical translation, for improved protocols to ensure reproducibility and scalability. Immune cell-derived EVs possess substantial therapeutic potential in cancer treatment, inflammatory diseases, and regenerative medicine. These tiny membrane-bound particles, naturally released by immune cells, carry bioactive molecules that can modulate immune responses, suppress tumor growth, or promote tissue repair. However, before these therapies can be widely used in clinics, key challenges must be addressed, particularly in standardizing their production, characterization, and quality control.

## Introduction

Extracellular vesicles (EVs) are membrane-bound structures released by different cell types into the surrounding extracellular environment [1–4]. These vesicles are essential intermediaries of intercellular communication, facilitating the transport of bioactive molecules, such as proteins, nucleic acids, and lipids [5–7]. EVs participate in both normal physiological and pathological conditions, regulating cellular functions and immune responses and contributing to disease progression [8]. EVs are typically categorized based on their size, origin, and mechanisms of biogenesis [9]. The primary category of EVs includes exosomes, typically ranging from 30 to 150 nm, which originate from the endosomal pathway and are released upon the fusion of multivesicular bodies (MVBs) with the cell membrane. Meanwhile, microvesicles (MVs), generally ranging from 100 to 1,000 nm, are formed through the outward budding of the plasma membrane [10, 11]. Another subtype of EV is apoptotic bodies (Abs), which range in size from 1 to 5 µm. Abs are formed during apoptosis, carrying cellular debris and contributing to immune regulation [12–14]. In the immune system, EVs play a crucial role in modulating immune responses by facilitating antigen presentation, transmitting cytokine signals, and enabling communication between immune cells [15–17]. They are crucial in regulating both innate and adaptive immune responses by modulating immune activation, suppression, and tolerance [18]. Their role in illnesses such as autoimmune disorders, cancer, and infections underscores their potential as therapeutic targets and diagnostic biomarkers [19,20].

The dual functionality of EVs derived from immune cells in immunomodulation and therapy presents promising opportunities in cancer treatment, inflammatory diseases, and regenerative medicine. However, unlocking their full therapeutic potential necessitates addressing several challenges, including elucidating their underlying mechanisms, establishing standardized protocols, and overcoming barriers to clinical translation. This systematic review seeks to examine the dual roles of immune cell-derived EVs in immunomodulation and therapy while identifying critical knowledge gaps and challenges related to their mechanisms, protocol standardization, and clinical application.

## Methods

### Literature search

**Figure 1.**
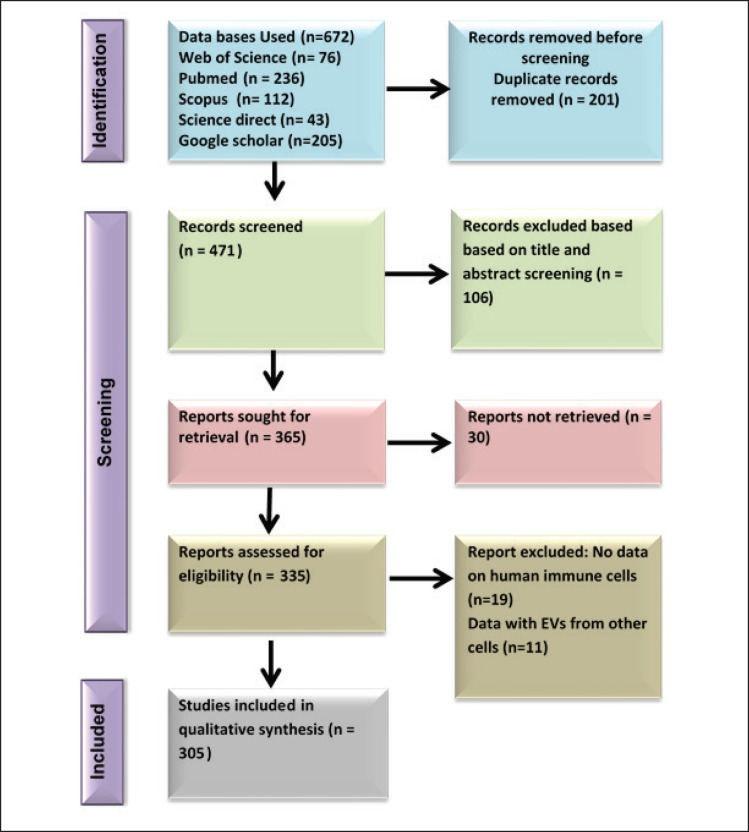
Summary of the procedure for identifying and selecting relevant.

To avoid redundancy and ensure no prior existence of the topic “The Dual Role of EV Derived from Animal and Human Immune Cells: A Systematic Review,” a comprehensive search was performed using various databases (PubMed, Scopus, Web of Science, Google Scholar, and ScienceDirect). Systematic searches, unrestricted by study date, language, or design, were initially conducted between October 2024 and December 2024, yielding 672 articles (Fig. 1). The articles searched were for the period between 2019 and 2024. The search strategy combined relevant keywords such as “EV,” “Immune Cells,” “Exosomes,” “MVs,” “Dual Role,” “Human Immune System,” and “Immune Modulation” using Boolean operators (“AND” and “OR”) and incorporated reference tracking for additional sources. Duplicate studies were excluded using Mendeley software, and articles were screened. The review of titles and abstracts was followed by a detailed evaluation of the full text. Inclusion criteria include: (a) studies involving immune cells (T-cells, B cells, macrophages, neutrophils, dendritic cells, basophils, eosinophils, and so on) from both human and animal models (mammalian species like mice, rats, and so on) and (b) studies focusing on immune responses and the role of EVs derived from these immune cells.

Exclusion criteria include: (a) studies not involving immune cells or those focusing on nonhuman (animal) models only, if the review aims to compare both human and animal models and (b) studies using irrelevant or nonmammalian species, unless focusing on comparative or cross-species analyses. However, gray literature, preprints, and non-English studies were excluded.

### Formation and development of EVs in immune cells of animals and humans

The production of EVs in animal and human immune cells involves complex intracellular transport mechanisms that are critical for their functional roles in immune regulation [21,22]. EVs are classified based on size, biogenesis pathways, and surface markers. According to MISEV2023 guidelines, EVs include exosomes (30–150 nm), originating from the endosomal pathway via MVBs, and MVs (100–1000 nm), formed by direct budding from the plasma membrane. A third category, Abs, is larger (500–2000 nm) and released during programmed cell death [22]. EV classification also relies on surface markers: exosomes typically express CD9, CD63, and CD81, while MVs may present Annexin V, integrins, or ARF6. The generation of EVs in immune cells occurs through specialized cellular processes. These vesicles are categorized based on their formation pathways, which mainly include the exosomal and MV pathways. These vesicles transport a variety of biomolecules that play essential roles in facilitating intercellular communication and regulating immune responses [23, 24]. Immune cells like dendritic cells and neutrophils release EVs that facilitate antigen presentation, lymphocyte activation, and enhancement of antimicrobial responses [25, 26]. The study of EV biogenesis remains an evolving field, highlighting the need for further research to fully understand their mechanisms and unlock their potential in immunotherapy [24, 26].

### Exosomal pathway

**Figure 2.**
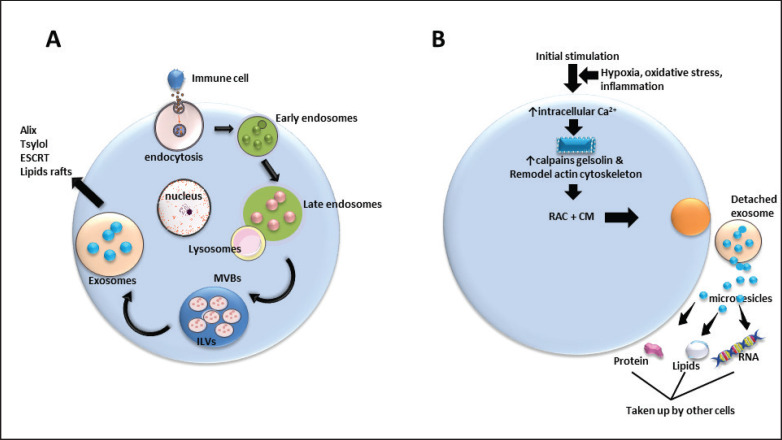
(A) Exosomal and (B) microvesicle biogenesis pathways. (A) The exosomal pathway involves the inward budding of the endosomal membrane, forming MVBs that fuse with the plasma membrane to release exosomes. (B) The microvesicle pathway entails direct outward budding of the plasma membrane, resulting in the release of microvesicles. Both pathways contribute to intercellular communication.

Exosomes are small EVs derived from the endosomal pathway that plays a key role in intercellular communication by transporting proteins, lipids, and nucleic acids [27–29]. Their formation begins with early endosomes, which mature into MVBs (Fig. 2A). These MVBs can either fuse with lysosomes for degradation or release their intraluminal vesicles as exosomes. The sorting of cargo into exosomes is primarily regulated by molecular mechanisms, particularly the endosomal sorting complexes required for transport (ESCRT) pathway [30]. Moreover, the uptake and cargo release mechanisms of exosomes depend on their source and the nature of the recipient cells, highlighting their intricate physiological functions [31]. Exosomes have gained a lot of interest because they can affect the environment around tumors and act as markers for diseases like neurodegenerative disorders. This highlights their promise for use in cancer treatment and diagnosis [27,30].

### MV pathway

MVs arise from the plasma membrane through outward budding and subsequent fission and are vital for intercellular communication and the modulation of diverse biological processes (Fig. 2B). These vesicles, which are larger than exosomes, carry a variety of molecular cargo, influencing the activities of recipient cells and potentially playing a role in the progression of the disease [32–34]. Their formation is closely tied to the lipid composition and biophysical characteristics of the plasma membrane, with specific lipid domains acting as sites for their generation [35]. MVs exhibit dual roles in cellular environments, affecting processes such as autophagy, apoptosis, and inflammation, which highlights their complex involvement in both maintaining health and driving disease [36]. Gaining insights into these mechanisms is essential for leveraging MVs in therapeutic strategies and diagnostic applications.

### Essential molecules in EV formation

The formation of EVs depends on various essential molecules and mechanisms that regulate cargo sorting and packaging. The ESCRT is crucial in the development of MVBs, which serve as precursors to exosomes, by facilitating the inward budding of endosomal membranes [37]. Tetraspanins, such as CD9, CD63, and CD81, are essential membrane proteins that help organize lipid microdomains and selectively incorporate proteins into exosomes [38]. RAB GTPases are also pivotal in transporting and fusing MVBs with the plasma membrane, allowing exosomes to be released into the extracellular space [37]. Specific lipids, such as ceramide and sphingolipids, contribute to membrane dynamics and EV stability, influencing their formation and cargo-loading processes [38]. Understanding these molecular components is crucial for executing the physiological and pathological roles of EVs in intercellular communication and their potential therapeutic applications [37] (Table 1).

**Table .** Key molecules involved in EV biogenesis.

S/n	Name of molecule	Function	Role in immune cells	Key proteins	References
1.	ESCRT	Involved in sorting cargo into ILVs Promoting membrane budding within MVBs	ESCRT complexes sort antigenic peptides into exosomes	TSG101 and ALIX	[37]
2.	Tetraspanins (CD9, CD63, and CD81)	Sorting of specific proteins and lipids into EVs	involved in immune cell activation modulate the composition of exosomes, influencing immune responses	MHC molecules co-stimulatory proteins necessary for antigen presentation	[38]
3.	RAB GTPases	Regulate vesicle trafficking, docking Fusion with the plasma membrane.	RAB27a facilitates exosome release in T cells	RAB27a mutations are associated with immunodeficiencies	[37]
4.	Lipids (Ceramide and Sphingolipids)	Formation of both exosomes and microvesicles	facilitate the transport of immune receptors and inflammatory mediators	promotes the packaging of proinflammatory signals into EVs	[38]

### Cargo of immune cell-derived EVs

EVs are crucial mediators of intercellular communication, transporting proteins, lipids, and RNAs that influence immune responses [39]. Their cargo varies with isolation techniques and includes immunomodulatory molecules like cytokines, chemokines, and lipids such as sphingomyelin, contributing to disease progression and therapeutic potential [40–42]. Immune cell-derived EVs carry regulatory elements, including miR-155 and miR-146a, which modulate macrophage polarization and T-cell activation in autoimmune and infectious diseases [28, 43, 44]. Long noncoding RNAs (e.g., HOTAIR) in EVs suppress immune surveillance by altering dendritic cell functions [44]. Engineered exosomes enhance cancer immunotherapy by delivering IFN-γ and granzyme B [44]. Their stability and low immunogenicity also support the delivery of siRNAs and mRNAs, advancing targeted therapies in immunological disorders [43, 44].

### Factors influencing EV release from immune cells

The production of EVs by immune cells is regulated by various factors, such as cell activation, intracellular calcium concentrations, hypoxia, stress, and interactions with pathogens (Table 2). Activation of immune cells, especially through immune stimuli, modifies the molecular composition of EVs, impacting their signaling functions and the assortment of small noncoding RNAs they transport [15]. Elevated intracellular calcium levels have been demonstrated to stimulate EV secretion, as shown by the identification of a small molecule that induces calcium influx, resulting in increased production of immunostimulatory EVs in dendritic cells [39]. In addition, hypoxic environments and cellular stress can affect the generation and release of EVs, showcasing the cell’s adaptive mechanisms to environmental pressures [40]. Interactions with pathogens further complicate this process, as EVs released during infections may contain bioactive molecules that facilitate antimicrobial responses, underscoring their significance in intercellular communication during immune challenges [41]. Collectively, these elements highlight the intricate and dynamic regulation of EV release in immune responses.

**Table 2.** Determinants of EV release by immune cells.

S/N	Factor	Impact	Mechanism	Example	References
1	Cell Activation	Activation of immune cells enhances the release of EVs	Cells upregulate vesicle formation pathways	B cells activated by antigen encounter release exosomes	[15]
2	Intracellular Calcium Levels	Elevated intracellular calcium levels are linked to increased microvesicle formation	Calcium influx disrupts membrane asymmetry	Platelet activation, which involves calcium signaling, results in the release of EVs enriched with clotting factors	[39]
3	Hypoxia and Stress	Stress significantly enhances the release of EVs	Hypoxia stabilizes hypoxiainducible factors (HIFs)	Tumor-associated macrophages under hypoxic conditions release exosomes carrying immunosuppressive factors, contributing to tumor immune evasion	[40]
4	Pathogen Interaction	Presence of pathogens can modulate EV release. Pathogen-derived molecules can be packaged into EVs	Pathogens like HIV or Mycobacterium tuberculosis can hijack the host cell’s EV	HIV-infected cells release exosomes containing viral proteins that can suppress immune cell activation, aiding in immune evasion.	[41]

### EVs derived from various types of immune cells

EVs derived from immune cells, such as SEVs (30–150 nm) and MVs (100–1000 nm), differ in size, origin, and surface markers. SEVs arise from MVBs, while MVs bud from the plasma membrane. Immune cells such as macrophages, dendritic cells, T and B lymphocytes, NK cells, and microglia produce EVs with unique markers (e.g., CD14, CD86, FasL, CD19, perforin, and Iba1) [42–44]. These EVs mediate immune responses and cell communication. Innate immune cells—macrophages, neutrophils, NK cells, dendritic cells, mast cells, and monocytes—provide rapid, nonspecific defense, while adaptive immunity involves B and T cells, which generate targeted responses and long-term memory [45, 46].

### T-cell-derived EVs

T-cell-derived EVs are vital mediators of immune communication, influencing both physiological and pathological processes [47]. Enriched with proteins, lipids, and nucleic acids, they regulate immune responses by modulating macrophage and dendritic cell activity [48]. These EVs transfer bioactive molecules, thereby shaping immune regulation [49]. T-cell-derived EVs exert immunomodulatory effects through various pathways. Apoptotic T-cell-derived EVs degrade cGAMP, suppressing the pro-inflammatory cGAS-STING pathway and alleviating radiation enteritis [45]. In pediatric COVID-19, plasma-derived EVs influence disease severity by altering T cell and monocyte activity [44]. Additionally, seminal plasma-derived EVs promote regulatory T-cell differentiation, enhancing immune tolerance [41].

Engineered EVs expressing immunomodulatory molecules such as CD80 and PD-L1 enhance or suppress T-cell responses [50]. In cancer, these vesicles modulate the tumor microenvironment, affecting tumor growth and immune evasion [51, 52]. Their therapeutic potential is promising, though challenges in isolation and clinical standardization persist [49–51]. T cells exhibit dual functionality, acting as pro-inflammatory mediators by secreting cytokines (e.g., IFN-γ, TNF-α) to enhance immunity or as immunosuppressors via regulatory T cells (Tregs), which inhibit excessive immune responses through IL-10 and TGF-β, maintaining immune homeostasis and preventing autoimmunity.

### B-cell-derived EVs

B-cell-derived EVs play a crucial role in immune responses by transferring proteins and nucleic acids to target cells, thereby modulating their function [52]. These vesicles enhance immune regulation by activating and differentiating T cells, strengthening pathogen defense mechanisms [53]. B-cell-derived EVs facilitate antibody transport, carrying immunoglobulin G (IgG) and immunoglobulin M (IgM). IgG+ EVs protect against influenza, while IgMcontaining EVs bind antigens and infiltrate target cells, enabling immune recognition [46, 48].

In cancer, B-cell-derived EVs influence the tumor microenvironment, promoting intercellular communication that may support tumor progression [47]. Clinically, they serve as therapeutic delivery vehicles, though their oncogenic signaling poses challenges [52]. Despite their promise, standardization of isolation and characterization remains a barrier to clinical application [52,53]. B cells display dual functionality, promoting inflammation by producing pro-inflammatory cytokines (e.g., IL-6, TNF-α) and autoantibodies while also exhibiting immunosuppressive roles through regulatory B cells (Bregs), which secrete IL-10 and TGF-β to suppress excessive immune responses, maintain tolerance, and prevent autoimmune diseases.

### Monocyte-derived EVs

Monocyte-derived EVs modulate immune responses in infections, cancers, and autoimmune diseases by transporting bioactive molecules that influence monocyte and macrophage behavior. Their effects vary between immune activation and suppression, depending on the context. Monocyte-derived EVs contribute to immune dysregulation in infections. In severe pediatric COVID-19, they promote immunosuppression by altering cytokine profiles and reducing monocyte and T-cell populations [50]. In malaria, EVs from *Plasmodium falciparum*-infected red blood cells influence monocyte polarization based on parasite virulence [49–51]. Similarly, in type 1 diabetes, stressed beta cell-derived EVs activate monocytes and exacerbate islet inflammation via pro-inflammatory markers [51].

In cancer, tumor-derived EVs drive monocyte differentiation into pro-tumor macrophages, fostering immune suppression and tumor progression [52, 54]. They also modulate tumor-immune interactions by altering cell activity [55, 56]. While monocyte-derived EVs hold therapeutic promise for targeted drug delivery, their dual role necessitates further study to mitigate tumor-promoting effects [57, 58].

### Natural killer cell-derived EVs

Natural killer cell-derived EVs (NKEVs) are promising therapeutic agents due to their cytotoxic properties, safety, low immunogenicity, and biocompatibility. They have potential applications in cancer and viral infections, making them valuable for clinical use. NKEVs enhance tumor infiltration by cytotoxic T cells, improving immune checkpoint inhibitor efficacy, especially in resistant tumors [42]. They exhibit direct cytotoxicity, reducing nonsmall cell lung cancer organoid viability by 40%–45%, potentially lowering chemotherapy doses [42]. However, low production yield and targeting efficiency necessitate further optimization [53].

In antiviral defense, NKEVs transfer microRNAs (miRNAs) that enhance immune responses, reducing SARSCoV-2 RNA levels and suppressing pro-inflammatory cytokines [52]. Their ability to function as safe nanomaterials supports their role as antiviral agents. Despite their potential, challenges in production efficiency and targeting persist. Engineering approaches are being explored to improve specificity and therapeutic effectiveness, highlighting the need for further research to optimize NKEV applications [52, 53]. NKEVs exhibit dual functionality, promoting inflammation by enhancing cytotoxic activity against tumors and infected cells or acting immunosuppressively by modulating immune responses through the transfer of miRNAs, which can dampen excessive immune activation, promoting immune tolerance in chronic conditions.

### Dendritic cell-derived EVs (DC-EVs)

DC-EVs enhance immune responses, making them valuable in cancer immunotherapy. These nanosized vesicles, particularly exosomes, are rich in major histocompatibility complex (MHC) molecules and co-stimulatory signals, effectively activating CD4+ and CD8+ T cells, key players in anti-tumor immunity [53,54]. Unlike tumor-derived EVs, DC-EVs do not carry immunosuppressive factors, making them ideal for therapeutic applications [55]. Engineered DC-EVs improve antigen presentation by delivering tumor-specific antigens like ovalbumin, boosting T-cell activation [56]. Their resistance to tumor-induced immunosuppression makes them superior to traditional dendritic cell vaccines, especially in digestive system cancers [53, 54]. They also enhance antigen transfer, promoting tumor-specific T-cell and natural killer cell activation [55].

Furthermore, DC-EVs regulate immune responses by modulating chemotaxis and inflammation [25]. However, challenges such as complex EV biology and limited clinical efficacy necessitate further research to optimize their therapeutic potential [55]. DC-EVs display dual functionality, promoting inflammation by activating T cells and enhancing immune responses through antigen presentation, or suppressing immunity by transferring immunosuppressive molecules (e.g., IL-10), fostering immune tolerance, and controlling excessive inflammation, particularly in autoimmune or chronic conditions.

### Neutrophil-derived EVs (NDEVs)

NDEVs are key regulators of immune responses and inflammation, influencing recipient cell function through bioactive molecules [43, 44]. These vesicles, including exosomes and ectosomes, are released during neutrophil activation or apoptosis. NDEVs play a dual role in cancer by either promoting or suppressing tumor progression through the transport of long noncoding RNAs and miRNAs, which modulate tumor biology and the extracellular matrix [57]. They also regulate immune homeostasis by modulating autologous neutrophil responses and inflammatory signaling, shaping the immune environment [59].

Beyond oncology, NDEs aid immune defense by transporting cytokines and chemokines, influencing immune cell behavior [25]. They also facilitate antigen presentation, enhancing immune activation and neutralizing virulence factors [25]. Despite their therapeutic potential, challenges in standardizing isolation and characterization limit clinical applications, necessitating further research into their roles in inflammation and immune regulation [44]. NDEs exhibit dual functionality, promoting inflammation by releasing cytokines, chemokines, and miRNAs that enhance immune responses or acting immunosuppressively by regulating neutrophil survival, modulating inflammatory pathways, and promoting tissue repair, thus balancing inflammation resolution and immune homeostasis in various conditions.

### Basophil-derived EVs

Basophil-derived EVs are key regulators of immune responses, particularly in allergic reactions and inflammation. Through piecemeal degranulation, basophils release vesicles containing histamine and bioactive molecules, enabling controlled immune modulation [60]. These EVs influence Th2 cell differentiation and enhance humoral memory, playing a crucial role in IgE-mediated allergic inflammation [61,62]. They also contribute to angiogenesis in allergic responses by expressing vascular endothelial growth factors, acting synergistically with mast cells to regulate inflammation [63].

Beyond allergies, basophil-derived EVs participate in immune modulation by transferring RNA and proteins that influence recipient cell function [64–68]. They are implicated in autoimmune and inflammatory diseases through the release of pro-inflammatory mediators [69–72]. Despite their rarity in circulation, their essential role in immune signaling highlights the need for further research into their therapeutic potential [73]. Basophil-derived EVs have dual functionality, promoting inflammation by releasing histamine and pro-inflammatory cytokines to enhance allergic responses or suppressing immunity by modulating immune cell activation and contributing to immune tolerance, particularly in allergic and autoimmune conditions, thereby balancing immune responses.

### Eosinophil-derived EVs

Eosinophil-derived EVs are crucial in immune modulation, particularly in allergic inflammation and eosinophilic disorders. Eosinophil sombrero vesicles (EoSVs) and exosomes, released during eosinophil activation and cytolysis, mediate intercellular communication and prolong inflammatory responses [74]. EoSVs persist in inflamed tissues, enhancing eosinophil-associated diseases (EADs) such as ulcerative colitis and eosinophilic chronic rhinosinusitis by modulating immune cell interactions [73–75]. Eosinophil-derived exosomes activate structural lung cells, promoting epithelial apoptosis and muscle proliferation in asthma [39]. Inflammatory stimuli further increase MV production, amplifying immune responses [40].

**Figure 3.**
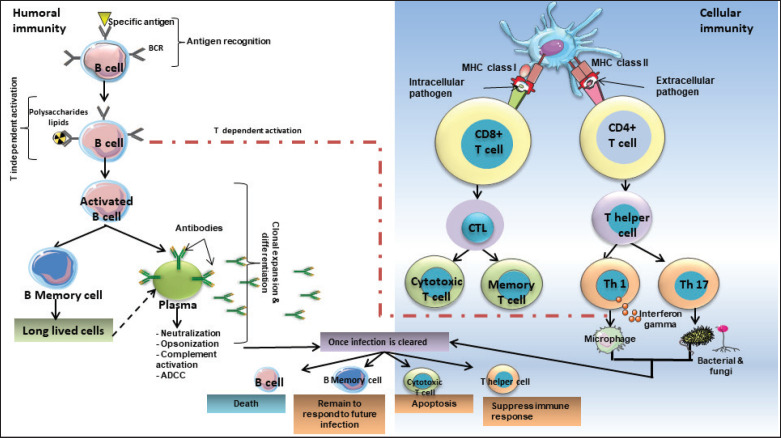
Coordinated defense mechanisms of humoral and cellular immunity. The diagram illustrates humoral and cellular immunity. Humoral immunity, driven by B cells, involves the production of antibodies that neutralize pathogens or mark them for destruction. Cellular immunity, mediated by T cells, includes helper T cells that activate immune responses and cytotoxic T cells that target and eliminate infected or abnormal cells. Both systems work synergistically, with helper T cells facilitating B-cell activation and cytotoxic T cells targeting infected cells. They provide a comprehensive defense against pathogens and abnormal cells, ensuring effective immune protection. ADCC: Antibody-Dependent Cellular Cytotoxicity, BCR: B-cell receptor, CTL: cytotoxic T lymphocyte, MHC I: major histocompatibility class I, MHC II: major histocompatibility class II, Th: T helper cells.

Due to their stability and immune-modulatory capabilities, EoSVs have been proposed as therapeutic agents for EAD treatment and drug delivery [75,76]. However, their heterogeneity and complex interactions necessitate further research to optimize their clinical applications (Fig. 3). EoSVs exhibit dual functionality, promoting inflammation by releasing cytokines and mediators that drive allergic responses and tissue damage or acting immunosuppressively by modulating immune cell activity, promoting tissue repair, and resolving inflammation, thus balancing immune activation and resolution in allergic and eosinophilic disorders.

### EVs derived from

EVs derived from microglia, key neuro-immune cells, mediate intercellular communication by transferring proteins, lipids, and RNAs. These EVs influence neuroinflammation, neuroprotection, and disease progression in neurodegenerative disorders. Their diagnostic and therapeutic potential underscores their importance in neuroimmunology and brain health research. Microglia-derived EVs exhibit both proand anti-inflammatory effects. Proinflammatory EVs carry cytokines (e.g., IL-1β and TNFα), promoting neuroinflammation and neuronal damage. Conversely, anti-inflammatory EVs deliver molecules such as IL-10, TGF-β, and miRNAs that suppress inflammation, support neuroprotection, and aid tissue repair, highlighting their dual role in central nervous system homeostasis and disease [77].

### Role of EVs derived from Immune cells in infection

**Figure 4.**
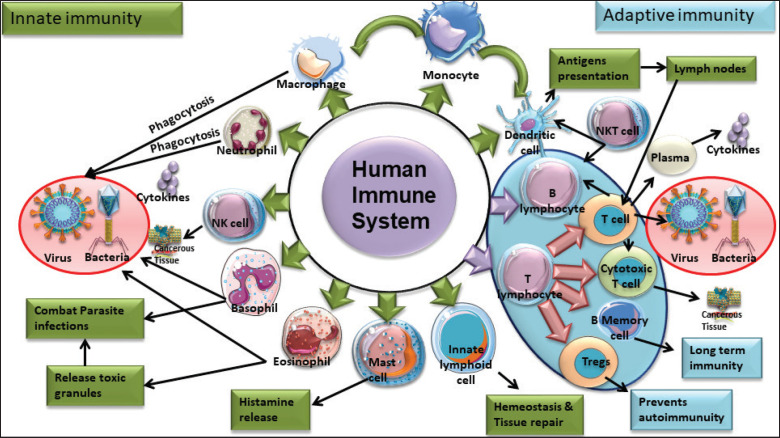
Role of immune cell-derived EVs in innate and adaptive immunity. The diagram depicts the dual functions of immunederived EVs in maintaining immune homeostasis. EVs contribute to immune regulation by dampening excessive responses, promoting tolerance, and supporting tissue repair, they also play a pivotal role in immune activation and amplification by transferring antigens and bioactive molecules to immune cells, enhancing signaling pathways, and strengthening adaptive immunity. These versatile roles underscore the importance of EVs in balancing immune responses and their implications for health and disease.

EVs derived from immune cells play a crucial role in infections by mediating intercellular communication and modulating immune responses. These vesicles, which carry a diverse array of biomolecules, can both enhance and suppress immune functions depending on the infection type. For instance, CD4+ T-cell-derived small EVs promote B-cell activation, proliferation, and antibody production, thereby enhancing adaptive immunity (Fig. 4) [78]. Conversely, pathogens can exploit EVs to facilitate their virulence; for example, viral infections can hijack exosome biogenesis to enhance viral spread, while bacterial EVs can manipulate host immune responses to promote infection [79]. Additionally, EVs from both immune and pathogenic cells influence the severity of lung infections, highlighting their dual role in disease pathogenesis [80]. Understanding these dynamics is essential for developing novel therapeutic strategies against infectious diseases [80].

### EVs in immune-related diseases

Immune-derived EVs are essential in the development and management of various diseases, especially those involving inflammation and immune dysfunction. These tiny vesicles, secreted by immune cells such as dendritic cells, T cells, and macrophages, enable communication between cells and help regulate immune system activity, influencing disease progression and therapeutic outcomes [41]. In cancer therapy, EVs can both stimulate and suppress immune responses, making them valuable for vaccine development and drug delivery systems [81]. In immune-mediated central demyelinating diseases like multiple sclerosis, EVs have emerged as promising candidates for biomarkers and therapeutic agents, with distinct profiles observed in affected individuals compared to healthy controls [82]. Furthermore, exosomal RNAs, including miRNAs, are emerging as critical regulators in immunological diseases, offering promising avenues for diagnostics and targeted therapies [83]. The therapeutic potential of EVs originating from immune cells underscores their significance in precision medicine and disease management.

### Dual roles of EVs derived from the immune system

EVs derived from the immune system play dual roles in immune regulation and activation, significantly influencing inflammatory responses and therapeutic strategies. Activated CD4+ T helper cell-derived EVs (act-EVs) enhance pro-inflammatory signaling in antigen-presenting cells, facilitating the recruitment of immune cells and the release of cytokines and thereby amplifying the immune response [84]. Engineered EVs derived from epithelial cells can be tailored to express immunomodulatory molecules, allowing them to deliver both activating and inhibitory signals to T cells. This capability is particularly significant in the context of cancer and autoimmune diseases [85–87]. The therapeutic promise of EVs originating from immune cells is further highlighted by their ability to modulate inflammation across various diseases, highlighting their promise in precision medicine [28]. Ultimately, the unique properties of EVs, including their lipid membrane structure and cargo delivery capabilities, position them as vital players in both immune regulation and therapeutic interventions [43, 88].

EVs derived from immune cells exhibit dual functionality, either promoting inflammation and immune responses or suppressing excessive activation to maintain homeostasis. This duality is context-dependent, with EVs playing contrasting roles in different microenvironments and diseases. For example, tumor-associated macrophage-derived EVs promote tumor progression and metastasis, while DC-EVs enhance anti-tumor immunity. Similarly, monocyte-derived EVs can either drive inflammation in acute settings or promote immune tolerance in chronic conditions like autoimmune diseases. NDEVs support infection resolution but may shift to immune suppression during tissue repair. The cargo carried by EVs, such as cytokines, miRNAs, and tumor antigens, determines their effects. In the tumor microenvironment, EVs from immune cells can activate cytotoxic T cells or contribute to immune evasion, depending on the stage of cancer. Understanding these mechanisms is essential for harnessing EVs in therapeutic applications, such as cancer immunotherapy and autoimmune disease management.

### EVs in immune suppression and tolerance

**Figure 5.**
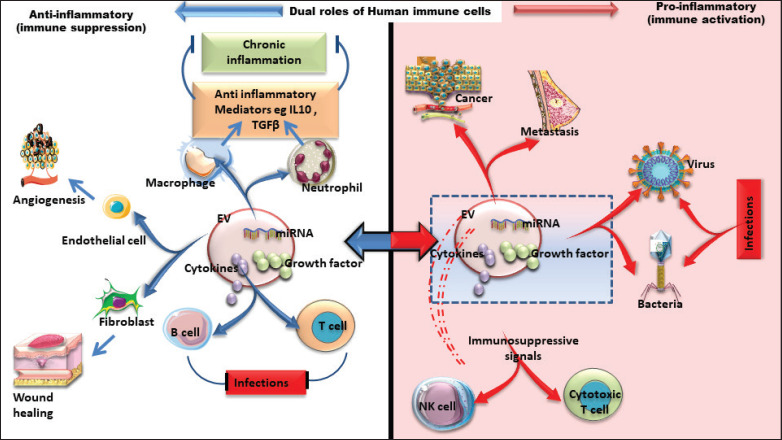
Dual roles of immune cell-derived EVs. This figure illustrates the dual roles of immune cell-derived EVs in immune modulation. Positive roles (blue color) include promoting immune activation by delivering antigens, cytokines, and co-stimulatory molecules to enhance pathogen recognition, adaptive immunity, and tissue repair. Negative roles (red color) involve suppressing immune responses by carrying inhibitory signals, regulatory molecules, or tolerogenic factors that dampen inflammation, support tumor progression, or facilitate immune evasion.

EVs play dual roles in immune suppression and tolerance. Specifically, their interactions with T regulatory (Treg) cells and dendritic cells (DCs) play a key role. EVs derived from Treg cells are essential for sustaining immunological tolerance by transmitting immunosuppressive signals, such as specific miRNAs, which can inhibit DC maturation and promote a tolerogenic environment [89,90] (Fig. 5). For instance, engineered small EVs loaded with miR-494 and miR-146a prevent DC maturation, reducing inflammatory responses [89].

Additionally, immunosuppressive MVs derived from tolerant DCs enhance Treg induction and promote anti-inflammatory macrophage polarization, effectively managing inflammatory diseases [91]. These mechanisms highlight the potential of EVs as therapeutic agents in controlling immune responses, particularly in autoimmune disorders and transplantation settings [25, 90].

### Mechanisms of action of EVs derived from the immune system

#### Signaling pathways involved

**Figure 6.**
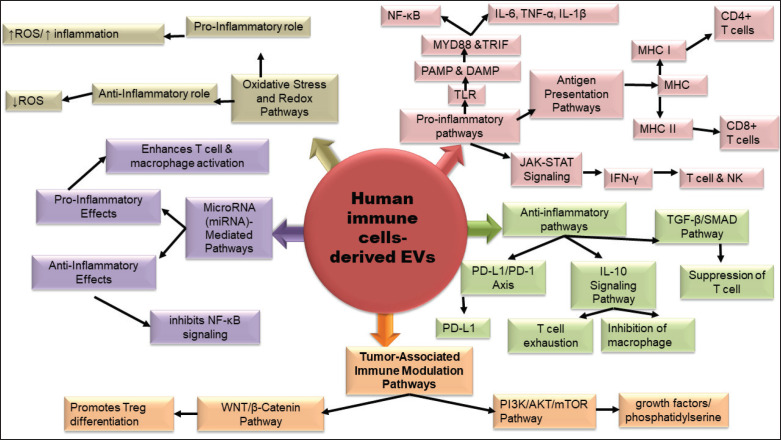
Signaling pathways involved in immune cell-derived extracellular vesicles. Immune cell-derived EVs exhibit dual roles in immune regulation by modulating pro-inflammatory and anti-inflammatory signaling pathways. They activate immunity via TLR/NF-κB, JAK-STAT, and antigen presentation pathways, promoting cytokine production and T-cell activation. Conversely, they suppress immunity through TGF-β/SMAD, PD-L1/PD-1, and IL-10 pathways, inducing Tregs and reducing inflammation. EVs influence the tumor microenvironment via PI3K/AKT/mTOR and WNT/β-catenin pathways, supporting tumor-promoting macrophages and suppressing cytotoxic T cells. Additionally, miRNAs (e.g., miR-155 and miR-146a) and redox molecules within EVs regulate oxidative stress and immune responses. These mechanisms highlight EVs’ potential for therapeutic modulation of immunity in diseases and cancers.

EVs from immune cells are critical mediators of immune regulation, facilitating intercellular communication by transporting bioactive molecules such as cytokines, MHC molecules, lipids, and RNAs [92,93]. For example, DC-EVs enhance adaptive immunity by presenting antigens via MHC-I/II and activating T cells through PI3K/AKT and NFAT pathways [92]. Similarly, macrophage-derived EVs stimulate inflammatory responses via NF-κB and MAPK signaling. Probiotic-derived EVs interact with TLR2 on host cells, activating JNK/MAPK and NF-κB pathways to induce cytokines like IL-6 and TNF-α, thereby strengthening innate immunity [94]. During infections, NDEVs enhance host defense by delivering antimicrobial proteins and activating the NLRP3 inflammasome and caspase-1, promoting IL-1β secretion [95,96]. In cancer, EVs show dual roles: TAM-derived EVs may support tumor progression via miR-21/STAT3 and PI3K/AKT pathways, while NK cell-derived EVs induce tumor cell apoptosis through granzyme B and caspase activation [92]. In kidney transplantation, donor-derived EVs drive alloimmunity by presenting antigens through TCR/NF-κB signaling, whereas MSC-derived EVs promote tolerance via TGF-β/SMAD signaling [39] (Fig. 6). However, immune cell-derived EVs exhibit diverse immunomodulatory functions, positioning them as promising tools for diagnostics and therapeutics across various disease states.

#### Interaction with target cells

EVs derived from immune cells play a crucial role in intercellular communication and immune modulation through various mechanisms. These lipid bilayer-enclosed particles, lipids, and transfer proteins, as well as nucleic acids, influence target cells by docking onto their membranes or entering via endocytosis and membrane fusion [28, 97]. The interaction of EVs with target cells modulates critical signaling pathways, impacting processes such as immune regulation, apoptosis, and antigen presentation, particularly in cancer and autoimmune diseases [43, 90, 97, 98] (Fig. 5). For instance, Treg cell-derived EVs create a tolerogenic environment by delivering specific miRNAs that regulate gene expression, thereby suppressing inflammatory responses [90]. This versatile strategy emphasizes the therapeutic promise of EVs in immunotherapy, showcasing their capacity to either amplify or suppress immune responses based on the specific context [98].

### Comparative roles of EVs in animals and humans

Research on EVs derived from human immune cells offers significant benefits for veterinary medicine, thanks to the shared characteristics between human and animal immune systems. EVs play a critical role in regulating immunity, controlling inflammation, and impacting disease development, providing veterinarians with tools to better understand immune responses in animals, including livestock, pets, and wildlife. These vesicles can be used to diagnose infections, boost immune function, and improve vaccine effectiveness by delivering antigens. Their ability to both promote and suppress inflammation makes them particularly useful for treating conditions such as equine asthma or canine inflammatory bowel disease. Additionally, EVs contribute to managing zoonotic diseases, identifying illnesses like mastitis or kidney disease, and aiding tissue repair in injuries or degenerative disorders. By applying insights from human EV research, veterinary diagnostics, treatments, and vaccine development are advancing, aligning with the principles of the One Health approach.

### Therapeutic applications of EVs derived from the immune system

EVs derived from immune cells have emerged as promising therapeutic agents in various medical fields, particularly in cancer and inflammatory diseases. These vesicles facilitate intercellular communication by transferring bioactive molecules, modulating immune responses, and influencing tumor progression [81]. Their dual role in promoting anti-tumor immunity while contributing to immune escape mechanisms underscores their potential in cancer therapy [74]. Additionally, EVs derived from stem cells, particularly mesenchymal stem cells, exhibit therapeutic advantages due to their lower immunogenicity and tumorigenicity, making them suitable for regenerative medicine and chronic conditions like diabetes [99]. The engineering of EVs for enhanced drug delivery and targeted therapy is a growing area of research, although challenges such as standardization and scalability remain [100]. EVs derived from immune cells represent a cutting-edge approach in precision medicine, with significant implications for future therapeutic strategies [100].

### EV as biomarkers of immune function

EVs have emerged as significant biomarkers of immune function, reflecting their critical functions in intercellular communication and immune modulation. They carry bioactive molecules, including nucleic acids, lipids, and proteins, which influence immune responses and disease pathology [25,101]. In cancer, EVs enhance antitumor immunity and address challenges like tumor heterogeneity [26]. Additionally, EVs are implicated in autoimmune diseases, where they present self-antigens and trigger immune responses, making them valuable for diagnosing conditions such as systemic lupus erythematosus and rheumatoid arthritis [102]. Their stability in body fluids and ability to convey information about the physiological state of cells underscore their potential as noninvasive biomarkers for monitoring immune function and disease progression [102,103].

### Technological advances in EV research

Technological advances in the study of EVs derived from immune cells have significantly enhanced their potential as therapeutic tools in immunology and cancer treatment. EVs, which are nanosized particles released by various cells, including immune and tumor cells, play crucial roles in cell communication and immune modulation [55]. Recent research highlights their ability to carry bioactive molecules, facilitating targeted delivery and enhancing the efficacy of immunotherapeutic agents, particularly in cancer [26, 104]. For instance, engineered EVs can display immunomodulatory ligands on their surfaces, improving signaling efficacy and shifting the tumor immune environment towards an anti-tumorigenic state [104]. Furthermore, immune cell-derived EVs show promise in treating inflammatory diseases by modulating inflammatory responses, representing a cutting-edge approach in precision medicine [26]. However, challenges such as standardization of isolation methods and understanding their biological mechanisms remain critical for their clinical translation [26].

### Advantages and limitations of EVs derived from immune cells

EVs derived from immune cells play crucial roles in modulating immune responses and show promise for therapeutic applications. T, B, dendritic, and NK cell-derived EVs regulate adaptive and cytotoxic immunity, while monocyte, neutrophil, eosinophil, and basophil EVs target inflammation and allergic responses. With high biocompatibility and the ability to cross biological barriers, these EVs offer engineering potential for drug and vaccine delivery. Diagnostic biomarkers such as CD markers, cytokines, and enzymes enhance their utility. However, challenges include limited yield, stability, and targeted delivery in some EV types (Table 3).

**Table 3.** Advantages of immune cell-derived EVs.

Type of immune cell-derived extracellalar vesicles	Immunomodulatory properties	Targeted delivery	Biocompatibility and low immunogenicity	Crassing Biological barriers	Potential for engineering	Diagnastic biomarhers	Natural antibody carriers	Inflammatory response regulation	References
T-cellderived EVs	Modulate T-cel activation, immune suppression, and cytokine signaling	Target T cells and antigenpresenting. cells (APCs)	Highc derived from hast immune system	Cross endothelial and trikgee barriers	Enginaerad to deliwer therapeutic RNAS or peptides tangeting immure disorders	CD3, CD4, CDB, TCRs, and cytokines	Potentially carry T-cellspecific antibodies	Regulate adaptive immune responses, including T-cell activation and suppression	[105, 106]
B-cellderived EVs	Present antigens to T cells; regulate humoral immunity	Deliver antigens to *T* cells	High immune tolernce with minimal side effects	Cross thasue barriers to target immure niches	Engineering for antigen delivery and vaccine development	CD19, CD20, CD40, and Ig molecules	Natural carriers of antibodies	Influence numoral immune responses and B-cell activation pathways	[46, 107]
Monocytederived EVs	Promote macrophage differentiation; cytokine production	Target inflammatory situes and macrophages	High effective in inflammatory microervironments	Crows endothelial and thisase barriers	Engliseered for inflammatory diseases and tissue regeneration	CD14, CD16, and miRNAs	Rarely antibody carriers	Regulate inflammatory respontaes via cytokines [ 0.8 . [l-1, TNF-a]	[50]
Natural killar cellderived EVs	Cytotosic actluity against tumor and infected cells; release perforing and granzymes	Specific targeting of tumor or virally infected cells	High, low immunogenicity in host tissues	Cross vascular barriers to reach infected or cancerous sites	Engineered for cancer immunotherapy and antiviral strategies	Perforin. granzyme, and NK markers (CD56 and CD16)	Not direct carriers	Regulate innate immune cytotoxicity and inflammatory signaling	[42, 52]
Dendritic cell-derived Evs	Antigen presentation and T-cell activation	Target T cells, inducing immane responses	High, role in imemunolagical tolerance	Cross Iymphatic and vascular barriers	Engineering for vaccine delivery and cancer immunotherapy	MHC molecules, CD11c, and costimulatory molecules	Not direct carriers	Regulate both adaptive and innate immune responses	[53-55]
NDEV	Pro-inflammatory cytokine release; NET formation	Target inflammatory and infection situes	Highc derived from innate immune cells	Crass vascular and triase barriers	Potential for targeted delivery in infections and inflammatory diseaves	MPO , neutrophil elastase, miRNAs	Rarely antibody carriers	Regulate acute inflammation and pathogen clearance	[43, 44]
Basophilderived EVs	Histamine and cytokine signaling: IgE-mediated allengic responses	Limited targeting specificity	Moderate; role in allergic responses	Limited ability to cross barriers	Emerging interest in allergy modulation and immune response enginearing	Histamine, IL-4, and IL-13	Not direct carriers	Regulate IgE-mediated Inypersensitivity reactions	[60-62]
Eosinaphilderived EVs	Modulate parasitic defense; proinflammatory in allengic diseasies	Limited targeting specificity	High; associated with allengr and parasitic deferse	Cross thasue barriers in allengic and parasitic contexts	Engineering for allergy, asthma, and paracitic dikeases	Essinophil perosidase and MBP	Not direct carriers	Regulate allergic inflammation and chronic inflammatory responses	[39, 70]
Mast cellderived EVs	Histarnine, cytokine, and pratease release in allengic and inflammatory responses	Target inflammatory and allengyassociated situs	Highc mediate strong allergic and imeviane responses	Cross vascular and tisloye barriers	Potential for allengy management and immurse response engineering	Histamine, tryptiase, and TNF$\alpha$	Not direct carriers	Regulate allengic inflammation and acute immune responses	[108, 109]
Innate Iymphaid cell-derived Evs	Cytokine release; regulation of innate and adaptive immune responses	Limited but can target specific immure cells	High low immunogenicity in innate immune conterts	Crass tiksow and vascular barriers	Potential for therapy in inflammatory and autoimmune diseases	Cytakines (e.g. IL-S, IL-13, and |FN-y|	Not direct carriers	Regulate tissue homeostasis, inflammation, and innate immunity	[110]

Furthermore, immune cell-derived EVs show great potential for therapeutic applications but face challenges in isolation, heterogeneity, yield, and circulation stability. While EVs from T cells, B cells, NK cells, and DCs offer targeted delivery and immune modulation, their low yield and short half-life hinder their use. Neutrophil-, basophil-, eosinophil-, and mast cell-derived EVs are involved in inflammation and allergic responses, but their instability and immune activation risks complicate therapeutic use. Engineering specific cargo and understanding EV mechanisms remain major hurdles in exploiting their full potential for disease management (Table 4).

**Table 4.** Limitations of immune cell-derived EVs.

Type of immune cellderived EVs	Complex isolation and purification	Heterogeneity of EVs	Limited yield	Short half-life in circulation	Potential for unwanted immune activation	Storage and stability challenges	Difficulty in engineering specific cargo	Limited understanding of mechanisms	References
T-cell derived EVs	Complex due to similarity with other lymphocyte EVs	Highly heterogeneous in size and cargo composition	Moderate; depends on T-cell activation	Short due to rapid clearance by phagocytic cells	Potential due to pro-inflammatory cytokine cargo	Susceptible to aggregation and degradation at high temperatures	Challenging to load specific RNA or protein cargo	Limited understanding of T-cell EV roles in immune modulation	[111, 112]
B-cellderived EVs	Challenging; requires separation from circulating antibodies	Heterogeneous; include antibody-laden and antigenloaded EVs	Low; B-cell EV production is less abundant	Short, especially in noninflammatory environments	Risk of activating offtarget immune responses	Stability varies based on antigen/cargo composition	Difficulty in maintaining antigen stability during loading	Incomplete understanding of mechanisms in humoral immunity	[113, 114]
Monocytederived EVs	Difficult due to overlap with macrophagederived EVs	Highly variable depending on activation state	Low to moderate yield	Rapid clearance in circulation	Risk of triggering inflammation or cytokine storms	Prone to degradation under suboptimal conditions	Cargo engineering is complicated due to diverse monocyte functions	Mechanisms of immune modulation not fully elucidated	[28, 113, 115]
Natural killer cellderived	Complex due to small quantities and shared markers with T cells	Heterogeneous; Low due to enriched limited NK in perforin, cell numbers	Low due to limited NK cell numbers	Short; quickly cleared by phagocytes	Potential for cytotoxic effects on nontarget cells	Highly sensitive to environmental conditions	Loading granzyme-like therapeutic molecules is challenging	Limited understanding of EV-mediated cytotoxic pathways	[116, 117]
Dendritic cellderived EVs	Complex; requires extensive antigen profiling	Highly diverse, reflecting the antigenic diversity	Low; limited production per dendritic cell	Short unless modified for longer circulation	May inadvertently suppress or overactivate immune responses	Require cryopreservation; degrade rapidly at room temperature	Engineering precise antigenic cargo is difficult	Mechanisms for T-cell activation by EVs are still being explored	[112]
NDEVs	Challenging due to overlap with NET structures	Heterogeneous; carry inflammatory and antimicrobial molecules	Moderate during acute inflammation	Very short; quickly cleared after release	Risk of exacerbating inflammation in certain conditions	Highly unstable in storage; degrade rapidly	Specific cargo engineering limited by fast production kinetics	Understanding of EV functions in infection control is incomplete	[73, 118]
Basophil-derived EVs	Difficult due to low cell counts and yield	Limited diversity; mainly histamine and cytokines	Very low yield	Very short; low circulation stability	Potential to trigger allergic or hypersensitivity reactions	Unstable under standard storage conditions	Engineering precise histamine-related cargo is underexplored	Mechanisms of EV-mediated allergic responses not fully understood	[73]
Eosinophilderived	Challenging; overlap	Heterogeneous; linked to	Low yield during	Short in circulation	Potential for exacerbating	Highly unstable; susceptible to	Limited ability to	Limited data on EV roles	[119, 120]
Mast cellderived	Difficult due to	Heterogeneous; rich in	allergic responses	Short due to clearance by tissue-resident cells	Risk of inducing hypersensitivity or anaphylaxis	Require specific storage conditions for stability	Engineering histamine or proteaserelated cargo is complex	Mechanisms of EV-mediated mast cell communication are underexplored	[121, 122]
Innate lymphoid cellderived $\mathrm{E} V \mathrm{~s}$	Difficult; low abundance in tissues	Limited diversity; predominantly cytokinerelated	Very low due to scarcity of innate lymphoid cells	Short due to limited stability in circulation	Potential for triggering unintended inflammatory responses	Highly unstable outside controlled environments	Cargo engineering limited by lack of molecular targets	Incomplete understanding of their roles in tissue homeostasis	[123-125]

### Future perspectives and challenges

The future of immune cell-derived EVs is promising yet faces significant challenges. EVs, particularly those derived from DCs, serve as vehicles for cancer immunotherapy, especially in colorectal cancer, by delivering bioactive molecules that activate tumor-specific immune responses. However, the immunosuppressive nature of tumor-derived EVs complicates their therapeutic application; nonetheless, they have demonstrated promise in boosting immune reactions, and engineering strategies are being developed to further improve their ability to stimulate immune responses. Consequently, probiotic-derived EVs are emerging as modulators of host immune responses, indicating broader applications in health and disease management. Despite these advancements, significant hurdles remain, including the need for standardized isolation methods, a more profound understanding of EV biology, and the integration of findings from wildlife studies to enrich immunological research. Overcoming these obstacles will be essential to fully harness the therapeutic capabilities of EVs in clinical applications.

## Conclusion

Current research on EVs in immunology highlights their dual role in cancer therapy and wild immunology, revealing both therapeutic potential and challenges. EVs derived from immune cells, particularly DCs, are recognized for their ability to enhance immune responses by delivering antigens without the immunosuppressive cargo typical of tumor-derived EVs, thus showing promise in cancer immunotherapy. Moreover, EVs enable interaction between cancer stem cells and immune cells, shaping the tumor microenvironment and potentially contributing to mechanisms of immune evasion. In the broader context of immunology, EVs are emerging as valuable tools for studying immune responses across diverse species, addressing logistical challenges in wildlife research. Despite these advancements, the clinical application of EV-based therapies remains limited due to issues such as incomplete understanding of EV biology and standardization in isolation methods. Emerging technologies like single-EV analysis and CRISPR-modified EVs are advancing EV research. Clinical trials, such as NCT04313647, investigate the use of mesenchymal stem cell-derived EVs for treating COVID19, showcasing their potential in targeted therapies and therapeutic applications in immune modulation and tissue repair. Future research is essential to unlock the full potential of EVs in both cancer treatment and broader immunological studies.

## List of abbreviations

ALIX, ALG-2-interacting protein; Ca2+, calcium; CM, cytoplasmic membrane; ESCRT, endosomal sorting complexes required for transport; EVs, extracellular vesicles; IFN-γ, interferon-gamma; IL, interleukin; ILVs, intraluminal vesicles; JAK-STAT, Janus kinase/signal transducers and activators of transcription; MHC, major histocompatibility complex; miRNA, microRNA; MVBs, multivesicular bodies; NF-κB, nuclear factor kappa-light-chain-enhancer of activated; NK, natural killer cells; PD-L1/PD, programmed death-ligand 1; PI3K/AKT/mTOR, phosphatidylinositol-3 kinase/protein kinase B/mechanistic target of rapamycin; RAC, receptor activator of calcineurin; ROS, reactive oxygen species; TGF-β/SMAD, transforming growth factor-β/small mother against decapentaplegic; TLR, toll-like receptor; TSG101, tumor susceptibility gene; WNT/β-catenin, βcatenin-mediated WNT signaling.
